# Identifying disease associations via genome-wide association studies

**DOI:** 10.1186/1471-2105-10-S1-S68

**Published:** 2009-01-30

**Authors:** Wenhui Huang, Pengyuan Wang, Zhen Liu, Liqing Zhang

**Affiliations:** 1 Department of Computer Science, Virginia Tech,2050 Torgerson Hall, Blacksburg, VA 24061-0106, USA; 2 Program in Genetics, Bioinformatics, and Computational Biology, Blacksburg, VA 24061-0106, USA

## Abstract

**Background:**

Genome-wide association studies prove to be a powerful approach to identify the genetic basis of different human diseases. We studied the relationship between seven diseases characterized in a previous genome-wide association study by the Wellcome Trust Case Control Consortium. Instead of doing a horizontal association of SNPs to diseases, we did a vertical analysis of disease associations by comparing the genetic similarities of diseases. Our analysis was carried out at four levels – the nucleotide level (SNPs), the gene level, the protein level (through protein-protein interaction network), and the phenotype level.

**Results:**

Our results show that Crohn's disease, rheumatoid arthritis, and type 1 diabetes share evidence of genetic associations at all levels of analysis, offering strong molecular support for the current grouping of the diseases. On the other hand, coronary artery disease, hypertension, and type 2 diabetes, despite being considered as a natural group with potential aetiological overlap, do not show any evidence of shared genetic basis at all levels.

**Conclusion:**

Our study is a first attempt on mining of GWA data to examine genetic associations between different diseases. The positive result is apparently not a coincidence and hence demonstrates the promising use of our approach.

## Background

Human genomes differ only in about 0.1% from each other, but this small genomic difference contains the key difference that can determine a person's susceptibility to diseases. In order to identify the genomic basis of certain diseases, genome-wide association (GWA) studies, an approach to find genetic variations (e.g. single nucleotide polymorphisms – SNPs) associated with a particular disease have become increasingly popular and useful. With completion of the Human Genome Project and HapMap Project and availability of dense genotyping chips and assembly of large and well-characterized clinical samples [1], it is now technically possible and financially feasible to conduct GWA studies that are powerful to detect candidate genes for certain genetic diseases. Meanwhile, the surging amount of available GWA data provides us an excellent opportunity for mining of disease relationships.

In this study, we focused on understanding the genetic basis of associations between seven common human diseases, using the data generated by a recent extensive GWA study undertaken in the British population [2]. The study examined about 2,000 humans for each of seven major diseases and a shared set of about 3,000 controls. This study was led by the Wellcome Trust Case Control Consortium (WTCCC) that brought together over 50 research groups from the UK that are active in researching the genetics of common human diseases. The seven diseases examined are bipolar disorder (BD), coronary artery disease (CAD), Crohn's disease (CD), hypertension (HT), rheumatoid arthritis (RA), type 1 diabetes (T1D), and type 2 diabetes (T2D). Although these seven diseases differ in their clinical symptoms, according to the WTCCC [2], theses diseases can be clustered into three natural groups: CAD+HT+T2D (metabolic and cardiovascular phenotypes with potential aetiological overlap); RA+T1D (already known to share common loci); and CD+RA+T1D (all autoimmune diseases). However, whether the grouping has sound genetic basis, that is, whether the diseases that belong to the same group share similar genotypes, was not addressed in depth in the WTCCC study. Elucidating the genetic commonality between diseases (i.e. whether different diseases are caused by some common loci) can help us discover possible hidden relationships between diseases that may appear unrelated phenotypically. It may also improve therapeutic treatment, disease diagnosis, and better prevention [3].

In this study, we took advantage of the GWA data of the seven diseases to examine whether different diseases share some level of commonality in genotypes. Our goals are to: (1) fish out sets of SNPs associated with the seven diseases in the WTCCC study and analyze whether there are overlaps between different sets of SNPs that correspond to different diseases, (2) analyze commonalities between genes associated with the SNPs in these diseases, (3) construct protein-protein interaction networks for the sets of genes and explore common features of the networks across the diseases, and (4) analyze the phenotypic similarities between the diseases.

## Results and discussion

### Analysis of SNP clusters

The GWA study by the WTCCC produced a list of SNPs that are associated with each of the seven diseases. The confidence of association of a SNP with a specific disease is represented by the SNP's P-value. The lower the P-value is, the more likely that the SNP is associated with the disease. Similar to the WTCCC study [2], we discarded the SNPs that have P-value higher than 10^-4 ^because these SNPs are weakly associated with the diseases and are more likely owing to some statistical noise than to real biological significance. The SNPs with *P*-value = 10^-4 ^were clustered into blocks. There are around 100 clusters (blocks) of SNPs for each disease, with blocks that are on the same chromosome at least 1 MB (mega base pairs) apart from each other.

**Figure 1:**
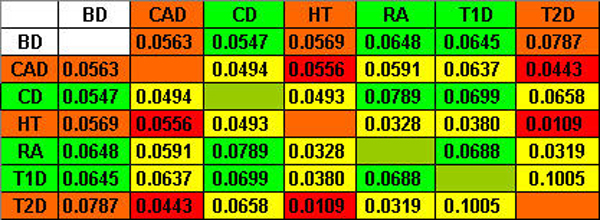
Jacobian value matrix.

In order to measure the degree of commonalities between SNP blocks of different diseases, we employed two different metrics, the Jaccard index and the total length measurement. For both metrics, the larger the metric is, the more similar two cluster patterns are to each other. First, figure 1 shows a matrix of Jacobian values between each pair of seven diseases. It is a symmetric matrix. According to the disease grouping by the WTCCC (see Background), we colored diseases into red groups (CAD, HT, T2D) and green groups (CD, RA, T1D). We expect to see more similarities between diseases within the same group, and thus cells with deep red and deep green are expected to be bigger than others in the same row or column. This proposition is true for the green group. However it is not true for the red group.

**Figure 2:**
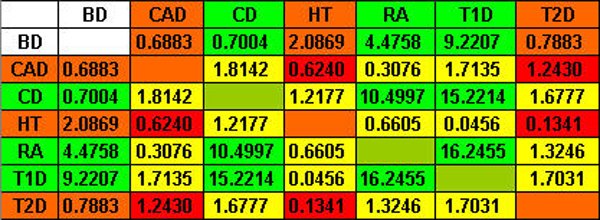
Total length matrix.

Second, we also used the total length to measure similarities between two sets of clusters. Figure 2 shows the matrix for seven diseases. Similar to the results based on the Jacobian values, strong similarities are observed in the green group (CD, RA, T1D), and are not observed in the red group (CAD, HT, T2D).

Therefore, results on both measurements of the similarities in SNP blocks for disease pairs show that the grouping of CD, RA, and T1D has a strong genetic basis, whereas the grouping of CAD, HT, and T2D has little genetic evidence. Within the green group (CD, RA, and T1D), it is unclear which two of them have a higher degree of commonality. For example, the Jocobian value between CD and RA is the highest within the three diseases, whereas the total length measurement between RA and T1D is the largest.

**Figure 3:**
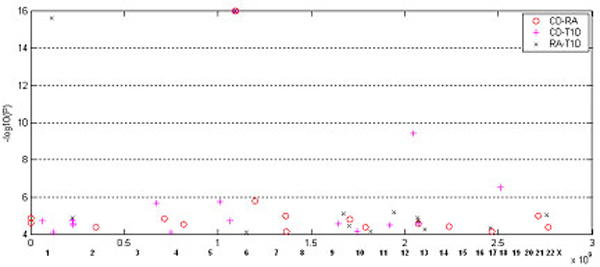
Distribution of cluster pairs for CD, RA, and T1D. The y axis value -*log*_10 _*P *is the average of paired clusters' strongest P-values.

Since strong correlations in SNP blocks are observed between CD, RA, and T1D, one would wonder whether paired blocks/clusters between these three diseases have highly similar distribution patterns. We plotted three sets of paired clusters between these three diseases in figure 3. If paired clusters are the same, then they should appear at the same location, which is not the case. Therefore, although there are strong genetic associations between each pair of the three diseases, the patterns of such relations are quite different from each other and there is little commonality shared by all three diseases.

### From SNPs to genes

As some of the SNP clusters reside in genes that may have different biological functions [4,5], we can map the SNP clusters to genes for each disease and characterize the degree of association between diseases at the gene level. Studying the pattern of gene overlaps between different diseases will allow us to move one step further towards the functional mechanisms of diseases, most of which result from abnormality or absence of certain proteins in the human body. Thus not only would we be able to obtain a clearer picture than from the SNP clusters in how diseases may be related, but also it facilitates our investigation of disease association at the level of protein networks [6].

**Figure 4:**
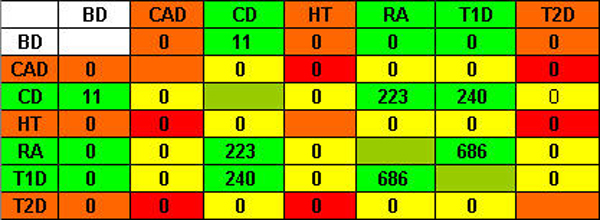
Numbers of shared genes under 0 distance.

**Figure 5:**
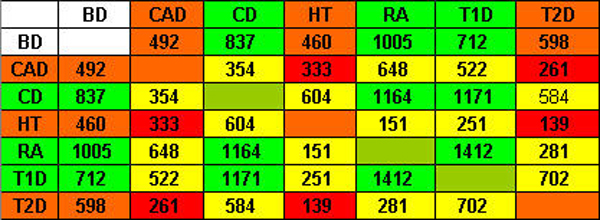
Numbers of shared genes under maximal distance.

The number of candidate genes shared between diseases can indicate association of diseases. The more candidate genes shared by two diseases, the more closely related between the two diseases [7]. Thus, when SNP blocks are converted to genes, we expect to see the relation between any two diseases by calculating the number of shared genes. As stated in the methods, we examined the distribution of the distances of SNP blocks to their downstream genes and decided to use the two extreme values for this analysis. Figures 4 and 5 display the comparison results on the number of shared genes between all possible combinations of disease pairs with 0 distance (i.e. the SNP blocks must overlap with genes) and the maximal distance (i.e. all SNP blocks are converted into genes). We observe a large number of genes shared between RA, T1D, and CD, which are all auto-immune diseases. However, the other pre-categorized group CAD, HT, and T2D, does not show any significant relation within its three members. Therefore, the results of the analysis at the level of genes display similar outcomes to the analysis at the level of SNPs.

2 KB upstream regions of genes tend to be enriched with regulatory elements [7]. SNPs in such regions as well as in transcription factor binding sites (TFBSs) are mutations that might affect the regulation of gene expression and genetic interactions. Interestingly, we found that although there are 266 SNPs in RA, for example, located within the 2 KB upstream region of genes, none of them reside in the predicted regions of TFBSs. Thus, there is little evidence for the claim that some studied SNPs may lead to abnormal regulation of gene expression that in turn contributes to the pathogenesis of diseases.

### From genes to protein networks

Analysis of SNPs can show us the low level of association between primary sequences and diseases. But in order to understand the disease pathogenesis from a functional point of view, we need to study SNPs (and thereof genes) at the protein level because the majority of genes must be translated into proteins to participate in protein-protein interaction networks or pathways and to perform their biological functions [6]. Mapping gene data onto the protein-protein interaction (PPI) network and comparing the PPI networks associated with different diseases will allow us to investigate whether different diseases have overlaps in networks or pathways, and thus may provide an alternative approach for identifying the genetic basis of commonalities or associations between diseases.

**Figure 6:**
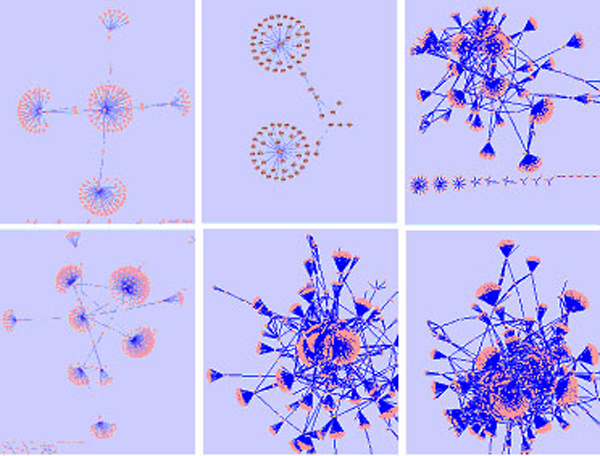
the PPI networks for: from left to right, BD, HT, RA (upper panel), CAD, CD, and T1D (Lower panel), respectively.

Using the STRING database, we constructed seven disease networks. Figure 6 shows the seven networks, each of which contains a set of proteins that are associated with the diseases. Because hub nodes form the backbone of a network, they are considered to be an important measurement for the similarity between protein interaction networks; moreover, backbone network has also been shown to be highly conserved in maintaining housekeeping biological function of the cell [6]. Thus, we examined all the hub proteins in the networks to see whether there are shared hub proteins. We defined hub proteins as the protein nodes with degrees ≥ 15 in a disease network [6]. Table 1 shows the number of hub proteins identified for each disease. The T1D has the highest number of hub proteins among the seven diseases, probably due to the fact that it has the highest number of genes that were used to construct the disease network.

**Table 1:** Number of hub proteins in each disease.

Disease	Number of hub proteins
BD	2

CAD	11

CD	21

HT	2

RA	17

T1D	25

T2D	0

Shared hub protein analysis reveals two findings (Table 2). First, CD, RA, and T1D shared a protein, the DNA-directed RNA polymerase I subunit provides the molecular evidence for the grouping of these three diseases. It has been shown that the DNA-directed DNA polymerase activity of RA specimens was increased in the high-speed pellet fraction of cell lysates, which makes virus infection in RA more vulnerable [8]. Also it has been shown that polymerase chain reaction is a very important step in Mycobacterium paratuberculosis, which is in turn a infectious cause of Crohn's disease [9]. The DNA-directed RNA Polymerase I subunit can be easily exploited by virus to produce virus DNAs for subsequent infection. We were not able to find any literature documenting the connection between the DNA-directed RNA Polymerase and T1D. Therefore, existing literature seems to support our finding of the molecular link – the DNA-directed RNA Polymerase, for the associations between at least two of the three diseases. Second, RA and T1D share five proteins (Table 2), suggesting that they have a stronger association in their molecular mechanisms of pathogenesis as compared to CD and T1D or CD and RA. Table 3 shows the biological function of the five proteins and whether their association with RA and T1D is supported by existing literature. Four proteins were found to be associated with RA and two proteins with T1D. Two proteins, HLA class I and IEX-1, are both supported to have association with RA and T1D. Also notice that HLA class I has immune response function, which is consistent with the grouping of CD, RA, and T1D into auto-immune diseases.

**Table 2:** The number of shared hub proteins in disease networks.

Diseases	Number of hub proteins
CD, RA, and T1D	1

RA and T1D	5

**Table 3:** The function of five hub proteins shared by RA and T1D and supporting publications.

Protein name	Function(GO)	Association with RA	Association with T1D	Pubmed id
HLA class I histocompatibility antigen	Immune response	Yes	Yes	11369787

Radiation-inducible immediate-early gene IEX-1	NOT found in GO	Yes	Yes	16368886, 14630199

Collagen alpha	Integral to membrane; Beta-amyloid binding; Heparin binding	Yes	No	8816431

Death domain-associated protein 6	Nucleus; Protein binding; Protein homodimerization acitivity; Transcription factor binding	No	No	n/a

Mediator of DNA damage checkpoint protein 1	Nucleus; Protein binding	Yes	No	17913746

Therefore, our results show that it is possible to identify the underlying molecular mechanism that might contribute to the commonality between different diseases, either in terms of pathogenesis or disease phenotypes, by tracing shared hub proteins between diseases.

### Comparison of disease phenotypes

All three levels of analysis of the seven diseases show consistent results that support the grouping of CD, RA, and T1D, but not the grouping of CAD, HT, and T2D. In order to further verify our results, we compared the phenotypes of the seven diseases to see how they are related at the phenotypical level using MimMiner. MimMiner is a phenotype comparison tool that uses the Online Mendelian Inheritance in Man (OMIM) database and various text mining algorithms to classify phenotypes [7]. The MimMiner score measures the degree of similarity of various diseases to the target disease in terms of phenotypes. The higher the score is, the more similar the two diseases are in terms of their phenotypes. Thus, we can judge using the MimMiner score the degree of similarity of various diseases to the diseases of our interest. Table 4 shows the MimMiner results for three of the seven diseases (for brevity, only the top ten hits for each disease were shown). We did not find any significant hits for BD, CAD, HT, and T2D from any of the seven diseases. It is clear that consistent with the results of the analysis performed at the levels of SNP, genes, and proteins, Crohn's disease, rheumatoid arthritis, and type 1 diabetes also have a close phenotypic connection, whereas coronary artery disease, hypertension, and type 2 diabetes do not.

**Table 4:** Disease names and their top 10 significant phenotype hits, the rows in bold are diseases of interest.

Rank	id	Score	Disease Name
Crohn's disease and its phenotype hits.			

1	266600	1.0000	inflammatory bowel disease 1

2	191390	0.6624	ulcerative colitis

3	605225	0.6402	inflammatory bowel disease 7

**4**	**180300**	**0.4314**	**rheumatoid arthritis**

5	177900	0.4256	soriasis susceptibility

6	301000	0.4220	wiskott-aldrich syndrome

**7**	**222100**	**0.4065**	**diabetes mellitus, insulin-dependent**

8	249100	0.4054	familial mediterranean fever

9	232220	0.4048	glycogen storage disease ib

10	219700	0.4043	cystic fibrosis

rheumatoid arthritis and its phenotype hits.			

1	180300	1.0000	rheumatoid arthritis

2	180350	0.4476	rheumatoid nodulosis

**3**	**266600**	**0.4314**	**inflammatory bowel disease 1**

**4**	**222100**	**0.4117**	**diabetes mellitus**

5	106300	0.3881	ankylosing spondylitis

6	191390	0.3861	ulcerative colitis

7	606044	0.3816	sjogren syndrome

8	254500	0.3736	myeloma, multiple

9	109100	0.3681	autoimmune disease

10	300310	0.3665	agammaglobulinemia

type 1 diabetes and its phenotype hits.			

1	125480	1.0000	diabetes mellitus

2	275000	0.4704	graves disease

3	601318	0.4478	diabetes mellitus

4	270150	0.4458	sjogren syndrome

5	600496	0.4415	maturity-onset diabetes of the young

6	217000	0.4141	complement component 2 deficiency

**7**	**180300**	**0.4117**	**rheumatoid arthritis**

**8**	**266600**	**0.4065**	**inflammatory bowel disease 1**

9	137100	0.4049	immunoglobulin a defi1

10	125850	0.4042	maturity-onset diabetes

## Conclusion

We analyzed associations between GWA studies of seven diseases. Our study was carried out at four levels – analysis at the nucleotide level (SNPs), analysis at the gene level, analysis at the protein level (protein-protein interaction network), and analysis at the phenotype level. For one group of diseases (CD, RA, and T1D), strong associations are found across all levels of analysis. In particular, our results indicate that within this group, RA and T1D are more strongly associated than they are to CD, suggesting RA and T1D are originated by highly similar molecular mechanisms, which can shed light on further exploration of these diseases. For another group of diseases (CAD, HT, and T2D), no genetic association is found at all levels of analysis. The negative result could be due to inappropriate grouping of the diseases (for example, classifying according to metabolic and cardiovascular phenotypes might be superficial and inaccurate) or that our analysis might be too primitive to recover the hidden genetic associations for this group. However, given our rather thorough analysis to uncover the relationships among these diseases and the strong confirmation of the grouping of CD, RA, and T1D, we think that the latter is highly unlikely.

Our study is a first attempt on mining of GWA data to examine genetic associations between different diseases. The positive result is apparently not a coincidence and hence demonstrates the promising use of our approach. With the increasing amount of GWA data, it becomes increasingly important to examine whether there are any hidden relationships between different diseases, especially the diseases without apparent phenotypic commonalities (e.g. similar disease symptoms). At the same time, many diseases have highly similar symptoms such that it remains controversial whether they are different diseases or actually one disease with different names. For example, there has been a long-standing controversy whether schizophrenia and bipolar are one or two disease traits [10]. It is conceivable that with proper data, our approach could make a definite contribution to this question and questions of similar kind.

## Methods

### Analysis of SNP clusters

In order to explore the relationship between SNPs of different diseases, we clustered SNPs into blocks and examined whether there are commonalities between SNP blocks of different diseases. The motivation for clustering SNPs into regions is that neighboring regions of the genome tend to have similar expressions and likely function together, by analyzing the SNPs on the level of clusters and genomic regions rather than specific points, we are more likely to identify patterns of a larger scale. Specifically, we examined the distribution of the distances between all of the two nearest SNPs on the same chromosome, and found that using the 1 MB as a cutoff value for clustering SNPs into blocks (i.e. the distance between SNP blocks is greater than 1 MB) achieves a good balance between the total number of blocks produced and the cutoff distance. We also tried several hierarchical clustering methods and found that the results are highly similar to our straightforward clustering method (i.e. using a cutoff). Therefore, for the remainder of our work, we used the SNP blocks produced by the straightforward clustering method.

**Figure 7:**
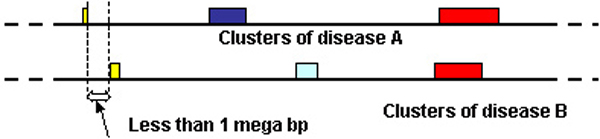
Demonstration of paired clusters. Same colored clusters are considered to be pairs.

To quantify the degree of commonalities between two diseases in terms of SNP blocks, we compared the distribution of the set of SNP blocks belonging to two diseases. We observed that some SNP clusters of different diseases share the same region or are physically very close to each other. We considered these SNP blocks to be paired blocks. Therefore, we can compare cluster patterns of different diseases by examining the prevalence of paired clusters. Figure 7 shows that two clusters from two diseases are paired when their locations on the chromosome overlap or are very close. The paired clusters demonstrate commonalities between the two sets of SNPs that are strongly associated with the two diseases.

We also use the total length to measure the similarity between clusters. The total length was calculated between each pair of sets of clusters, which is the sum of all paired clusters' lengths for two diseases (Figure 7). This value is proportional to the number of paired clusters and the cluster length (which indicates the reliability of the association). Thus we still expect larger value for stronger similarities.

### Conversion of SNP blocks to genes

When analyzing disease association at the SNP level, we in fact compared the distribution of SNP clusters between different diseases. At the level of genes, our task was to convert the SNP clusters to genes. Naturally, the first question was what kind of SNP clusters should be used. The confidence level of a SNP cluster was taken into account in our experiment. We selected SNP clusters by a threshold of P-value. Specifically, all SNPs having a P-value less than this threshold, i.e. with stronger relation to an individual disease above a lower bound, should be included into the clusters to be further converted into genes. We did several tests on different thresholds varying in a large range and set it to 10^-4^. We have two reasons for selecting this value. (1) SNPs with P-values less than this value are at least not insignificant. This P-value is the default threshold value used by the WTCCC for distinguishing moderate and significant SNPs from insignificant ones [2]. (2) Under this threshold, the numbers of genes converted and related to each disease seem reasonable. Table 5 shows that with threshold values of lower magnitudes than 10^-4^, the number of genes found for some diseases such as HT and T2D drops to 0, suggesting that the threshold values of lower magnitude than 10^-4 ^are too stringent.

**Table 5:** numbers of genes found for the seven diseases under different thresholds of P-value.

P-value	BD	CAD	CD	HT	RA	T1D	T2D
10^-4^	105	111	610	40	808	1095	33

10^-5^	3	9	43	0	618	994	5

10^-6^	0	2	22	0	584	956	0

10^-7^	0	1	14	0	584	857	0

### Construction of protein-protein interaction

First, from the seven sets of genes that are associated with the diseases, we translated the gene IDs into the ENSEMBL peptide IDs using the Biomart tool [11]. Second, we downloaded the STRING database [12], which contains all the known and predicted protein-protein interaction data and also direct (physical) and indirect (functional) associations. The strengths (scores) of protein-protein interactions and associations are evaluated by a composite criteria of multiple sources: Genomic Context, High-throughput Experiments, Conserved Co-expression, and Previous Knowledge that are mainly curated from the PubMed, and are thus quite robust.

### Determine upstream distance from genes

We used the human gene annotation in the UCSC's genome database for mapping SNP clusters to genes. This database contains adequate information about human genes and their locations along chromosomes. Before starting the conversion, the algorithm still remains unfixed for one issue. The straightforward method should work like the follows: if one SNP cluster shares a part of its region along the chromosome with a gene (or genes), the gene (genes) should be considered related to the cluster and added to the converted gene list. However this may not be a sufficiently sound criterion. In our experiment, we believe that we should take into account the regulatory effect of an SNP cluster from the upstream of a gene. This means that a cluster located upstream of a gene but having no shared regions sometimes can have a major effect on the gene, for example as a gene expression enhancer. In this case, when converting a cluster of SNPs to genes, not only genes having overlapping regions with the SNP cluster, but also genes located downstream of the cluster with no overlap, should be included into the converted gene list.

**Figure 8:**
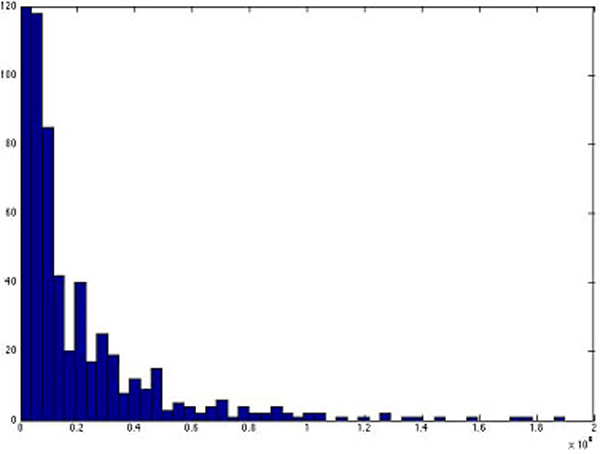
histogram for the distances of SNP blocks to the nearest genes.

We calculated the distances of all the SNP clusters to their nearest genes along chromosomes. Figure 8 shows how the distances of SNP clusters to nearest genes are distributed. Most cases (92.67%) of distances range from 0 to 500,000 base pairs. The highest frequency occurs in the range from 0 to 4,000 base pairs, which accounts for 15.88% of all cases, much higher than all the other ranges with the same width.

Since sometimes the regulatory region of a gene can be distantly located in the upstream region of the actual gene, it is hard to decide what distances should be used to convert SNP clusters into genes. We therefore used two extreme values for the distance allowed for a SNP block away from a gene: 0 and the maximum distance, to see the effect of distance on the conversion of SNP clusters to genes.

### From SNPs to genes analysis

SNPs in the regulatory regions of genes can have a major effect of the patterns of gene expression. In order to examine whether there are SNPs falling in regulatory regions of genes, we obtained more than 100,000 computationally identified transcriptional regulatory modules within the human genome from the PReMod database and wrote a C++ program to identify the SNPs that are within the transcriptional regulatory modules. The following describes the simple algorithm:

   **if **Extracting 2 KB upstream regions of genes. **then**

      **if **there are SNPs in the regions **then**

         **if **any TFBSs are inside the regions **then**

            Print result.

         **end if**

      **else**

            "No result"

      **end if**

   **end if**

## Competing interests

The authors declare that they have no competing interests.

## Authors' contributions

WH, PW, ZL designed the study, analyzed the data, and wrote the paper, LZ designed the study and wrote the paper.
